# Antibacterial Activity of Colloidal Silver against Gram-Negative and Gram-Positive Bacteria

**DOI:** 10.3390/antibiotics9010036

**Published:** 2020-01-19

**Authors:** Andrea Vila Domínguez, Rafael Ayerbe Algaba, Andrea Miró Canturri, Ángel Rodríguez Villodres, Younes Smani

**Affiliations:** 1 Clinical Unit of Infectious Diseases, Microbiology and Preventive Medicine, University Hospital Virgen del Rocío, 41013 Seville, Spain; andrea.vila.dguez@gmail.com (A.V.D.); ayerberafael@gmail.com (R.A.A.); amirocan93@gmail.com (A.M.C.); anrovi1797@gmail.com (Á.R.V.); 2 Institute of Biomedicine of Seville (IBiS), University Hospital Virgen del Rocío, CSIC, University of Seville, 41013 Seville, Spain

**Keywords:** multidrug-resistant bacteria, colloidal silver, Gram-negative bacteria, Gram-positive bacteria

## Abstract

Due to the emergence of antimicrobial resistance, new alternative therapies are needed. Silver was used to treat bacterial infections since antiquity due to its known antimicrobial properties. Here, we aimed to evaluate the in vitro activity of colloidal silver (CS) against multidrug-resistant (MDR) Gram-negative and Gram-positive bacteria. A total of 270 strains (*Acinetobacter baumannii* (*n* = 45), *Pseudomonas aeruginosa* (*n* = 25), *Escherichia coli* (*n* = 79), *Klebsiella pneumoniae* (*n* = 58)], *Staphylococcus aureus* (*n* = 34), *Staphylococcus epidermidis* (*n* = 14), and *Enterococcus species* (*n* = 15)) were used. The minimal inhibitory concentration (MIC) of CS was determined for all strains by using microdilution assay, and time–kill curve assays of representative reference and MDR strains of these bacteria were performed. Membrane permeation and bacterial reactive oxygen species (ROS) production were determined in presence of CS. CS MIC_90_ was 4–8 mg/L for all strains. CS was bactericidal, during 24 h, at 1× and 2× MIC against Gram-negative bacteria, and at 2× MIC against Gram-positive bacteria, and it did not affect their membrane permeabilization. Furthermore, we found that CS significantly increased the ROS production in Gram-negative with respect to Gram-positive bacteria at 24 h of incubation. Altogether, these results suggest that CS could be an effective treatment for infections caused by MDR Gram-negative and Gram-positive bacteria.

## 1. Introduction

Infections caused by multidrug-resistant (MDR) Gram-negative and Gram-positive bacteria such as *Acinetobacter baumannii*, *Pseudomonas aeruginosa*, *Enterobacter*, *Staphylococcus* spp., and *Enterococcus* spp. represent an increasing worldwide problem [1]. Currently, the appearance of MDR bacteria makes it impossible to find an effective drug to treat certain infectious diseases [2]. Therefore, there is an urgent need to find new therapeutic approaches in order to achieve better success in the bacterial infection treatment.

In this context, colloidal silver gained renewed interest. It was reported that colloidal silver can significantly reduce the duration and severity of many bacterial infections such as septic wounds [3]. This suspension of submicroscopic silver particles does not attack the bacteria directly, but causes a deactivation of enzymes responsible for their respiration, multiplication, and metabolism [4]. One of the main characteristics of silver is its oligodynamic effect, which is defined as the high microbicidal capacity of silver ions in water at a very low concentration (one part per million) [5]. Silver is an inert metal in its metallic form; however, it is biologically active when it is in the ionic monoatomic state (Ag^+^) soluble in an aqueous environment (water or tissue fluids) [6]. This activated ion shows a strong affinity for sulfhydryl groups and protein residues present in cell membranes [6]. 

Previous studies established four main mechanisms of action of silver ions: (i) destabilization of the cell membrane through the binding of silver ions to the sulfur atoms present in sulfhydryl groups of proteins and enzymes located on the bacterial cell surface [6,7,8], (ii) production of reactive oxygen species (ROS) [7,9], (iii) inhibition of metabolic pathways through the binding of silver ions to any protein that has some sulfur atom function [10], and (iv) interaction with bacterial DNA which causes the breakdown of cell cycle [11]. 

Recent studies reported that silver potentiates the antibacterial activity of ampicillin, ofloxacin, gentamicin, tetracycline, and chloramphenicol against *E. coli* in vitro and in animal models [7,12], tobramycin against biofilm producing *E. coli* and *P. aeruginosa* [13], and vancomycin against *E. coli* [7]. Moreover, silver was demonstrated to treat, as an adjuvant, persister cells which are tolerant to antibiotics [7]. Stable colloidal silver nanoparticles synthesized using *Caulerpa serrulate* displayed antibacterial activity at lower concentration against *S. aureus*, *P. aeruginosa*, *Shigella* sp., *Salmonella typhi*, and *E. coli* [14]. Of note, colloidal silver was reported to treat biofilm-related infections by *S. aureus* [15], methicillin-resistant *S. aureus*, and *P. aeruginosa* in vitro and in vivo in a model of *Caenorhabditis elegans* [16]. In clinical settings, colloidal silver was used topically for treatment of recalcitrant chronic rhinosinusitis, and it demonstrated a good safety profile with no major adverse events [17]. 

Despite these studies, there are some controversial reports on the antimicrobial activity of colloidal silver. The aim of this study was to determine the in vitro activity of colloidal silver against Gram-negative and Gram-positive bacteria, as well as to give clues on its mechanism of action.

## 2. Materials and Methods

### 2.1. Bacterial Strains

A total of 270 Gram-negative and Gram-positive references and clinical isolates were used in this study. The Gram-negative strains used were *A. baumannii* (*n* = 45), *P. aeruginosa* (*n* = 25), *E. coli* (*n* = 79), and *Klebsiella pneumoniae* (*n* = 58), of which clinical isolates of *A. baumannii*, *P. aeruginosa*, *E. coli*, and *K. pneumoniae* were MDR. The Gram-positive strains used were *S. aureus* (*n* = 34), *S. epidermidis* (*n* = 14), and *Enterococcus spp.* (*n* = 15), of which clinical isolates of *S. aureus* were methicillin-resistant, and clinical isolates of *S. epidermidis and Enterococcus* spp. were MDR. The reference strains of *A. baumannii* American Type Culture Collection (ATCC) 17978, *P. aeruginosa* O1 (PAO1), *P. aeruginosa* ATCC 27853, *E. coli* ATCC 25922, *K. pneumoniae Colección Española de Cultivos Tipo* (CECT) 997, and *S. aureus* ATCC 29213 were used. 

The strains of *A. baumannii* were isolated in the University Hospital of Virgen del Rocío of Seville (Spain) between 1998 and 2010 [18,19]. The strains of *P. aeruginosa* were isolated during the Spanish Group for Nosocomial Infections (GEIH)-bacteremia study between 2008 and 2009 [20]. The strains of *E. coli* and *K. pneumoniae* were isolated in the University Hospital of Virgen del Rocío of Seville (Spain) between 2016 and 2017 [21]. The strains of *S. aureus* and *S. epidermidis* were isolated in the University Hospital of Virgen del Rocío of Seville (Spain) between 2006 and 2007 [22,23]. The strains of the *Enterococcus* spp. were isolated in the University Hospital of Virgen del Rocío of Seville (Spain) during 2018.

### 2.2. In Vitro Susceptibility Testing

A stock solution of colloidal silver (10 ppm, PureSilver, Dusseldorf, Germany) was used for this study. Minimal inhibitory concentrations (MICs) of colloidal silver against Gram-negative and Gram-positive references and clinical strains were determined in two independent experiments by broth microdilution assay according to the EUCAST (European Committee on Antimicrobial Susceptibility European Committee on Antimicrobial Susceptibility Testing) recommendations [24]. The initial bacterial inoculum 5 × 10^5^ Colony Forming Unit (CFU)/mL for each strain was used in a 96-well plate (GreinerBioone, Germany) in the presence of colloidal silver, and incubated for 16–18 h at 37 °C. *P. aeruginosa* ATCC 27853 was used as a control strain. MIC_50_ and MIC_90_, which represent the concentrations shown to be effective for ≥50% and ≥90% of isolates tested, respectively, were determined.

### 2.3. Time–Kill Kinetic Assays

Time–kill curves of susceptible *A. baumannii* ATCC 17978, *P. aeruginosa* PAO1, *E. coli* ATCC 25922, *S. aureus* Sa24, and *E. faecalis* VS (vancomycin-susceptible) strains, with an MIC of vancomycin of 0.5 mg/L, and MDR *A. baumannii* #11, *P. aeruginosa* Pa238, *E. coli* Ec*mcr*1+ (*mcr*1-producing), *S. aureus* USA300#1 (clon USA300), and *E. faecalis* VR (vancomycin-resistant) strains, with an MIC of vancomycin of 128 mg/L, were performed in duplicate as previously described [25]. Initial inoculums of 1 × 10^6^ CFU/mL were conducted on Mueller Hinton broth (Sigma, Spain) (in presence of 0.5×, 1×, and 2× MIC of colloidal silver. Drug free broth was evaluated in parallel as a control. Tubes of each condition were incubated at 37 °C with shaking (180 rpm), and viable counts were determined by serial dilution at 0, 2, 4, 8, and 24 h. Viable counts were determined by plating 100 μL of control, test cultures, or the respective dilutions at the indicated times onto sheep blood agar plates (ThermoFisher, Spain). Plates were incubated for 24 h at 37 °C, and, after colony counts, the log_10_ of viable cells (CFU/mL) was determined. Bactericidal was defined as a reduction of ≥3 log_10_ CFU/mL with the initial inoculum.

### 2.4. Determination of Reactive Oxygen Species (ROS)

Fluorescent 2’,7’-dichlorofluorescin diacetate (DCFH-DA) (Sigma-Aldrich, Spain) was used to examine the levels of ROS. Initial inoculums of 1 × 10^6^ CFU/mL in minimal medium (M9) supplemented with glucose and magnesium sulfate were incubated, in the absence or presence of 0.25×, 0.5×, and 1× MIC of colloidal silver for each strain and control antibiotics (10 mg/L ampicillin, 10 and 64 mg/L ciprofloxacin, and 10 and 256 mg/L vancomycin) with 10 µM DCFH-DA in a 96-well plate, to determine the bacterial density and fluorescence using CLARIOstar Plus (BMGLabtech, GmbH, Germany). Bacterial density was determined at Optical Density 600 nm, and fluorescence was determined using excitation/emission wavelengths of 485/535 nm. Data were normalized to a no-dye control (background fluorescence) and OD_600nm_ (bacterial density) at 24 h of incubation. Twelve replicates per condition were measured in three independent experiments. The MICs of colloidal silver against ATCC 17978, #11, PAO1, Pa238, ATCC 25922, Ec*mcr*1+, *E. faecalis* VS, *E. faecalis* VR, Sa24, and USA300#1 were 4, 4, 4, 8, 4, 8, 8, 8, 8, and 8 mg/L, respectively.

### 2.5. Membrane Permeabilization Assay

Bacterial cells were grown in Luria Bertani Broth (Sigma, Spain) and incubated in the absence or presence of 0.25× MIC of colloidal silver for 24 h as previously described [26]. The pellet was harvested by ultracentrifugation at 4600× *g* for 15 min. Bacterial cells were washed with phosphate-buffered saline (PBS) 1×, and, after centrifugation in the same conditions described before, the pellet was resuspended in 100 µL of PBS 1× containing 10 μL of ethidium homodimer-1 (EthD-1) (ThermoFisher, Spain). After 10 min of incubation, 100 μL of mixture was placed into a 96-well plate to measure fluorescence at 0, 5, 10, 20, 30, 60, 90, 120, 240, and 300 min using a Typhoon FLA 9000 laser scanner (GE Healthcare Life Sciences, USA) and quantified by ImageQuant TL software (GE Healthcare Life Sciences, USA).

### 2.6. Statistical Analysis

Group data are presented as means ± standard errors of the means (SEM). The Student *t*-test was used to determine differences between means. A *p-*value < 0.05 was considered significant. The SPSS software, version 23.0 (IBM Corporation, Somers, New York, NY, USA) was used.

## 3. Results

### 3.1. Antimicrobial Activity of Colloidal Silver

Colloidal silver was tested against reference and clinical strains of *A. baumannii*, *P. aeruginosa*, *E. coli*, *K. pneumoniae*, *S. aureus*, *S. epidermidis*, and *Enterococcus* spp. The MIC_50_ and MIC_90_ concentrations, which were shown to be effective for ≥50% and ≥90% of isolates tested_,_ are presented in Table 1. The MICs for the Gram-negative bacteria strains ranged from 0.5 to >16 mg/L, while those for the Gram-positive strains ranged from 1 to >16 mg/L. The MIC_50_ and MIC_90_ for Gram-negative and Gram-positive strains ranged from 2 to 8 mg/L and 4 to 8 mg/L, respectively. These data show the antibacterial activity of colloidal silver. 

### 3.2. Time–Kill Curves

**Figure 1:**
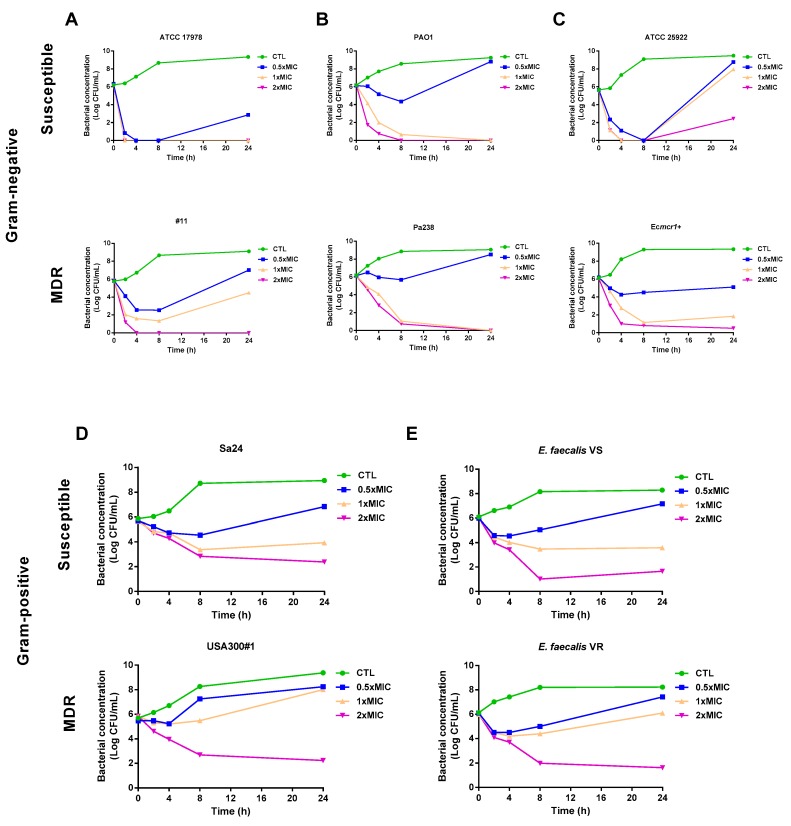
Colloidal silver presents bactericidal activity against Gram-negative and Gram-positive bacteria. Time–kill curves of susceptible and multidrug-resistant (MDR) strains of *Acinetobacter baumannii* (ATCC 17978 and #11) (**A**), *Pseudomonas aeruginosa* (PAO1 and Pa238) (**B**), *Escherichia coli* (ATCC 25922 and Ec*mcr1*+) (**C**), *Staphylococcus aureus* (Sa24 and USA300#1) (**D**), and *Enterococcus faecalis* (VS and VR) (**E**) in the presence of 0.5×, 1×, and 2× minimal inhibitory concentration (MIC) of colloidal silver for 24 h. CTL: control, bacteria without treatment. The MICs of colloidal silver against ATCC 17978, #11, PAO1, Pa238, ATCC 25922, Ec*mcr*1+, *E. faecalis* VS, *E. faecalis* VR, Sa24, and USA300#1 were 4, 4, 4, 8, 4, 8, 8, 8, 8, and 8 mg/L, respectively.

Using time course assays, we examined the bactericidal activity of colloidal silver against susceptible and MDR strains of *A. baumannii* (ATCC 17978 and #11), *P. aeruginosa* (PAO1 and Pa238), *E. coli* (ATCC 25922 and Ec*mcr1*+), *S. aureus* (Sa24 and USA300#1), and *E. faecalis* (VS and VR). Figure 1A shows that 0.5×, 1×, and 2× MIC of colloidal silver presented bactericidal effects against susceptible and MDR *A. baumannii* strains at 8 h decreasing the bacterial count by >3 log_10_ CFU/mL compared to the initial inoculum. These reductions persisted at 24 h at 0.5×, 1×, and 2× MIC of colloidal silver for the susceptible strain and at 2× MIC for the MDR strain. In the case of *P. aeruginosa*, 1× and 2× MIC of colloidal silver presented bactericidal effects against susceptible and MDR strains at 24 h (Figure 1B). Regarding *E. coli*, colloidal silver at 1× and 2× MIC were bactericidal against susceptible and MDR strains at 8 h. These bactericidal activities persisted at 24 h for 2× MIC of colloidal silver against the susceptible strain, and for 1× and 2× MIC against the MDR strain (Figure 1C). In the case of *S. aureus* and *E. faecalis*, only 2× MIC of colloidal silver presented bactericidal activity against susceptible and MDR strains at 24 h, although 1× MIC of colloidal silver was bactericidal against the susceptible *E. faecalis* strain at 24 h (Figure 1D,E). 

### 3.3. Colloidal Silver Effect on ROS Production

To assess whether colloidal silver caused an increase in cellular stress in the bacteria, the quantification of ROS was carried out by determining relative fluorescence units. The incubation of susceptible and MDR strains of *A. baumannii*, *P. aeruginosa*, and *E. coli* with 0.25×, 0.5×, and 1× MIC of colloidal silver increased progressively and significantly affected the production of ROS during 24 h when compared with untreated strains (Figure 1A–C). The positive controls (ampicillin and ciprofloxacin) also increased the production of ROS. 

**Figure 2:**
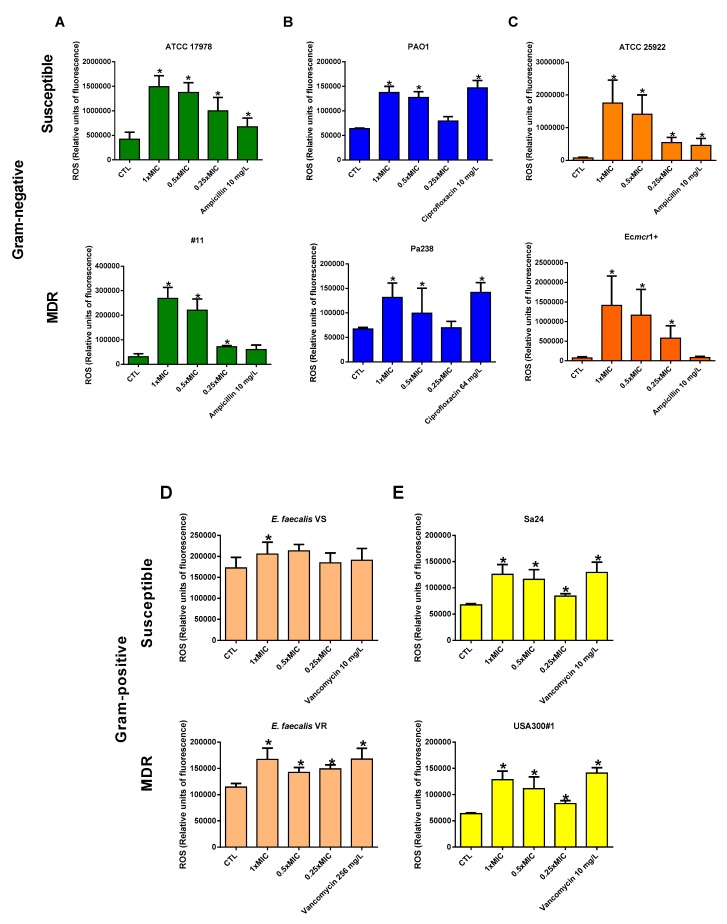
Production of reactive oxygen species (ROS) in Gram-negative and Gram-positive bacteria. Production of ROS by susceptible and MDR strains of *A. baumannii* (ATCC 17978 and #11) (**A**), *P. aeruginosa* (PAO1 and Pa238) (**B**), *E. coli* (ATCC 25922 and Ec*mcr1*+) (**C**), *S. aureus* (Sa24 and USA300#1) (**D**), and *E. faecalis* (VS and VR) (**E**) in the presence of 0.25×, 0.5×, and 1× MIC of colloidal silver, 10 mg/L ampicillin, 10 and 64 mg/L ciprofloxacin, and 10 and 256 mg/L vancomycin for 24 h. CTL: control, bacteria without treatment. Data are represented as means ± standard errors of the means (SEM) from 12 replicates in three independent experiments. * *p* < 0.05: treatment vs. control. The MICs of colloidal silver against ATCC 17978, #11, PAO1, Pa238, ATCC 25922, Ec*mcr*1+, *E. faecalis* VS, *E. farcalis* VR, Sa24, and USA300#1 were 4, 4, 4, 8, 4, 8, 8, 8, 8, and 8 mg/L, respectively.

Regarding *S. aureus* and *E. faecalis*, lower but significant changes in the production of ROS were observed under different concentrations of colloidal silver during 24 h, except for the vancomycin-susceptible *E. faecalis* (Figure 2D,E). The positive control (vancomycin) also increased the production of ROS. 

### 3.4. Colloidal Silver Effect on Bacterial Membrane Permeability

To evaluate whether colloidal silver is capable of causing any damage to the bacterial membrane, membrane permeability tests were performed. Susceptible and MDR strains of *A. baumannii, P. aeruginosa, E. coli, K. pneumoniae, S. aureus,* and *E. faecalis* were treated with 0.25× MIC colloidal silver and incubated with EthD-1. Five hours of fluorescence monitoring using a Typhoon scanner did not show an increase in the membrane permeability (data not shown). 

## 4. Discussion

The emergence of MDR Gram-negative bacteria prompted the use of colistin, as the last resort in the treatment of severe infections by these pathogens. Although uncommon, colistin resistance is increasing and its spread is being considered a global health threat.

Due to the disruptive action of silver on bacteria reviewed by Barras et al. and its ability to bind to the sulfur atoms present in sulfhydryl groups of proteins and enzymes located on the bacterial cell surface [4], we hypothesized that colloidal silver may present antibacterial activity against Gram-negative and Gram-positive bacteria. In this study, we showed that colloidal silver presented bactericidal activity with MIC_90_ values between 4 and 8 mg/L against a collection of 270 isolates of *A. baumannii*, *P. aeruginosa*, *E. coli*, *S. aureus*, *S. epidermidis*, and *Enterococcus* spp. These data are consistent with previous work reporting that colloidal silver was active against three reference strains of *A. baumannii*, *P. aeruginosa*, and *S. aureus* [27]. In other work, using Kirby–Bauer disc diffusion test, colloidal silver at 30 ppm was shown to be active against *S. aureus*, *S. epidermidis*, and *Bacillus subtilis* but not against *E. coli* [28].

It is noteworthy to mention that sub-MICs of colloidal silver did not significantly affect the membrane permeability of both Gram-negative and Gram-positive bacteria, in accordance with previously published data by Fenq et al. which showed that *S. aureus* is less permeable to silver ions when compared with *E. coli* [8]. In contrast, other studies showed that silver enhanced the cell permeability of a reference *E. coli* strain [7]. This difference in the membrane permeability results between both studies could be due to the fact that, in our study, we used MDR clinical isolates of *E. coli* that may have reduced membrane permeability. 

Although, in our study, the colloidal silver at sub-MIC against the studied strain could not alter the bacterial membrane permeability, colloidal silver may increase the production of ROS such as ampicillin [29], ciprofloxacin [30,31], and vancomycin [32]. Barras et al. reported in their review that silver is a non-redox active metal that cannot directly produce ROS [4]. The production of ROS by silver occurred through the perturbation of the respiratory electron transfer chain [9], Fenton chemistry following destabilization of Fe–S clusters, or displacement of iron [7], and inhibition of anti-ROS defenses by thiol–silver bond formation [33]. We observed in our study that colloidal silver at lower and higher concentration produced ROS in Gram-negative bacteria and to a much lesser extent in Gram-positive bacteria, especially in *E. feacalis*, which may explain the lower bactericidal activity of colloidal silver against Gram-positive bacteria in time–kill curves assays. Similar results were observed by Kim et al., who reported that the differences in structure, thickness, and composition of cells between Gram-negative and Gram-positive can explain why *E. coli* shows substantial inhibition by silver nanoparticles, whereas *S. aureus* is less inhibited [34]. The antimicrobial potential of silver ions is influenced by the thickness and composition of the cell wall of the microorganisms, and the difference in the organization of the peptidoglycan layer [35]. Gram-negative bacteria contain lipopolysaccharides (LPS) in the cell membrane, which contributes to structural integrity of the membrane, in addition to protecting the membrane from chemical attacks. However, the negative charge of LPS promotes the adhesion of silver and renders the bacteria more susceptible to antimicrobial therapy [35]. Several studies showed the pronounced adhesion and deposition of silver onto the cell surface of Gram-negative bacteria in particular, due to the presence of LPS in their cell membrane [36]. In Gram-positive bacteria, the cell wall is composed of a negatively charged peptidoglycan layer, and the amount of peptidoglycan is comparatively higher in Gram-positive bacteria than Gram-negative bacteria [35]. The lower susceptibility of Gram-positive bacteria to antibiotic therapy can be explained on the basis of the fact that their cell wall is comparatively much thicker than that of Gram-negative bacteria [37]. The thicker cell wall of Gram-positive bacteria, as well as the negative charge of the peptidoglycan layer, allows the adhesion of silver ions. For this reason, *S. aureus*, which possesses a thick cell wall and more peptidoglycan molecules, prevents the action of the silver ions and renders the bacterium comparatively more resistant to silver [8]. 

## 5. Conclusions

The results of this study provide new insights into the use of colloidal silver against MDR Gram-negative and Gram-positive bacteria, where the therapeutic options are reduced. Nevertheless, further studies are needed in order to elucidate the action of colloidal silver in vivo, as well as to determine the optimal dosage to achieve, in terms of efficacy and safety, clinical efficacy in the treatment of infections by MDR Gram-negative and Gram-positive bacteria.

## Figures and Tables

**Table 1:** Minimal inhibitory concentrations effective for ≥50% and ≥90% of isolates tested (MIC_50_ and MIC_90_) of colloidal silver for Gram-negative and Gram-positive bacteria.

Pathogen	*N*	MIC_50_ (mg/L)	MIC_90_ (mg/L)
**Gram-negative** *Acinetobacter baumannii*	45	4	4
*Pseudomonas aeruginosa*	25	2	4
*Escherichia coli*	79	2	8
*Klebsiella pneumoniae*	58	8	8
**Gram-positive**			
*Staphylococcus aureus*	34	4	8
*S. epidermidis*	14	4	4
*Enterococcus* spp.	15	4	4
